# Observations on Vegetable Anatomy

**Published:** 1802-06

**Authors:** Cit. Mirbel


### *Observations on Vegetable Anatomy, by* Cit. Mirbel.

(Extracted from a Memoir preſented to the National Inſtitute.)

ALL vegetables are compoſed of a membranous texture, the form and conſiſtency of which varies not only in the different ſpecies, but alſo in the ſame individual. There are no true fibres to be found in vegetables, the filaments to which that appellation has been aſſigned, being nothing but membranes, detached in ſmall longitudinal ſlips. The membranous texture, though cloſely coherent in all its parts, forms two ſpecies of different organs, viz. the cellular texture and the tubulous texture. The cellular texture conſiſts of a membrane, in the duplications of which interſtices, contiguous to one another, are hid, which when not compreſſed have an hexagonal figure, but by any preſſure from either ſide they receive the form of a parallelogram. The membranes of theſe cells are perforated with ſmall pores, through which the juices are to paſs. The exterior ſide of theſe cells is formed by the epidermis, which thus conſidered is not to be taken for a particular membrane. The cellular texture occurs in the fleſhy parts of plants, in the ſucculent fruits, the bark, the embryo, &c. The tubulous texture compriſes two kinds of tubes, the large ones and the ſmall ones. The large tubes are found in the cellular texture and occupy the centre of the ligneous part in the monocotyledones, and are ſometimes ſpread without order in the wood of the diatyledones; ſometimes however they are diſtributed in regular groupes and arranged in concentrical zones; they never occur in funguſſes, lichens, and fucuſſes. We may diſtinguiſh four ſpecies of large tubes. 1. The *simple* tubes, the ſides of which are without pores; they contain the ſucci proprii of plants, and are particularly numerous in the bark. 2. The *porous* tubes. Their ſides are perforated like a ſieve with many pores, which are arranged in regular and parallel rows; their uſe ſeems to be the ſame as in the firſt tubes. 3. The *falſe tracheæ*, (fauſſes trachées) are tubes tranſverſally interſected with parallel openings or porous tubes, in which the pores are larger than in the preceding ſpecies: They are moſtly found in wood that is not very hard, but particularly in the monocotyledones. 4. The *tra-*

*cheæ,*

*cheæ*. Thefe are tubes compofed of threads or filaments, which are turned in fpiral lines from the right to the left. They are obferved in all the foft parts of vegetables. The trachea of the butomus umbellatus, when once unfolded, do not contract again. The diftinction of thefe four fpecies of tubes, however, is by no means fo exact as to admit no exception; thus the butomus offers, in the fame tube, the pores of the porous tubes, the openings of the falfe trachea, and the fpiral windings of the true trachea. Thefe tubes Cit. Mirbel diftinguifhes by the name of *tubes mixt*.

The fmall tubes are compofed of cells united with one another, which are clofed at their extremities; their walls are frequently provided with pores; in the embryo they are not yet evolved. We may obferve them in fome lichens. Placed round the large tubes they make the ligneous filaments in the monocotyledones; and in the dicotyledones, being arranged round the marrow and the large tubes, they compofe the ligneous ftrata. They frequently fill and obftruct the large tubes. Cit. Mirbel gives the name of *lacunae* (lacunes) to regular and fymmetrical interftices, which are formed in the interior of vegetables by the dilaceration of the membranes, a phenomenon which is particularly obferved in plants of a lax texture. In the horfe-tail (equifetum), they are of an extraordinary regularity; one being larger than the reft, forms a tube in the centre of the ftalks, while two rows of fmall ones furround the central tube; in the leaves of the monocotyledones the lacunae are interfected with partitions vifible to the naked eye, confifting of cellular fubftances. It is probable the large tubes originate from the lacunae. Though there are no glands to be perceived in vegetables, they may be fuppofed to exift in the membranes, as fap is fecerned and elaborated in them. It is not improbable, the opacous wreaths that furround the pores and the orifices of the large tubes, are of a glandulous nature. The pores are fmall openings in the membranes, of which three fpecies may be diftinguifhed:—1. The *insensible* pores, which cannot be perceived, but feem to be the organs of the infenfible tranfpiration. 2. The *elongated* pores, or the organs defcribed by Decandolle, by the name of cortical pores, each of which correfponds to a cell; they ferve for the tranfpiration and abforption of fluids; their feat is the epidermis of the herbaceous part. 3. The *glandulous* pores; thefe are openings furrounded with opacous irregular wreaths; they occupy the interior, and fometimes the exterior, parts of plants. They are either very minute, or larger, in which cafe they feem to be formed by the union of the fmaller ones.—All parts of vegetables are originally mucilaginous; thus the embryo is, at the firft fight, nothing thing but a mucilage, very fimilar to the white of an egg. This mucilage is remaining at the interior bark (alburnum), and at the medulla in the dicotyledones, and it is placed round the ligneous filaments in the monocotyledones. The cellular as well as the tubulous texture feem to originate from this mucilage. The embryo adheres to the mother plant by means of an umbilical cord, which has a peculiar organifation. In this manner Cit. Mirbel endeavours to explain the vegetable organifation, but he obferves, at the fame time, how comparatively inconfiderable the progreffes are which we have hitherto made in the knowledge of the vegetable organifation.

